# GC–MS based antioxidants characterization in *Saussurea heteromalla* (D. Don) Hand-Mazz by inhibition of nitric oxide generation in macrophages

**DOI:** 10.1038/s41598-024-60577-7

**Published:** 2024-05-02

**Authors:** Saboon, Asia Iqbal, Yamin Bibi, Tayyiba Afzal, Ahmad Sher, Abdul Qayyum, Muhammad Akmal, Hesham S. Almoallim, Mohammad Javed Ansari, Yawen Zeng

**Affiliations:** 1 Department of Botany, Women University Mardan, Mardan, 23200 Pakistan; 2 Department of Wildlife and Ecology, University of Veterinary and Animal Sciences, Ravi Campus, Pattoki, Pakistan; 3 Department of Botany, Rawalpindi Women University, Rawalpindi, 46300 Pakistan; 4 Institute of Environmental Biology, Department of Plant Biology, Wroclaw University of Environmental and Life Sciences, ul. Kozuchowska 5b, PL 51-631 Wroclaw, Poland; 5 Institute of Agronomy, Bahauddin Zakariya University, Multan, 60800 Pakistan; 6 Department of Agronomy, The University of Haripur, Haripur, 22620 Pakistan; 7 Institute of Soil and Environmental Sciences, PMAS-Arid Agriculture University Rawalpindi, Rawalpindi, 46300 Pakistan; 8 Department of Oral and Maxillofacial Surgery, College of Dentistry, King Saud University, PO Box-60169, 11545 Riyadh, Saudi Arabia; 9 Department of Botany, Hindu College Moradabad (Mahatma Jyotiba Phule Rohilkhand University Bareilly), Bareilly, 244001 India; 10 Biotechnology and Germplasm Resources Institute, Yunnan Academy of Agricultural Sciences/Agricultural Biotechnology Key Laboratory of Yunnan Province/Key Laboratory of the Southwestern Crop Gene Resources and Germplasm Innovation, Ministry of Agriculture, Kunming, 650205 China

**Keywords:** ROS, RAW 264.7, Nitric oxide inhibition, *Saussurea heteromalla*, Biological techniques, Plant sciences

## Abstract

For centuries, medicinal plants have served as the cornerstone for traditional health care systems and same practice is still prevalent today. In the Himalayan region, *Saussurea heteromalla* holds a significant place in traditional medicine and is used to address various health issues. Despite its historical use, little exploration has focused on its potential for scavenging free radicals and reducing inflammation. Hence, our current study aims to investigate the free radical scavenging capabilities of *S. heteromalla* extracts. The *n*-hexane extract of entire plant revealed promising activity. This extract underwent extensive extraction on a larger scale. Subsequent purification, employing column chromatography, HPLC–DAD techniques, led to the identification of active compounds, confirmed via GC–MS and the NIST database as 1-O-butyl 2-O-octyl benzene-1,2-dicarboxylate and 2,4-ditert-butylphenol. Assessing the free radical scavenging properties involved utilizing RAW-264.7 macrophages activated by lipopolysaccharides. Notably, the compound 2,4-di-tert-butylphenol exhibited remarkable scavenging abilities, demonstrating over 80% inhibition of Nitric oxide. This study stands as the inaugural report on the isolation of these compounds from *S. heteromalla*.

## Introduction

Nitric oxide (NO) is an important signaling molecule synthesized from l-arginine by three forms of nitric oxide synthase (NOS), namely endothelial Nitric Oxide synthase (eNOS), neural Nitric Oxide synthase (nNOS) and inducible Nitric Oxide synthase (iNOS)^1^. In contrast to the others two, iNOS produce large amount of NO. NO plays an important role in host defense response against various pathogens and regulate various pathophysiological processes such as vasodilation, neurotoxicity, and neuronal communication^2^. However, the overproduction of NO cause severe toxicity leading to cell death and tissue damage results in acute or chronic inflammation. Some researchers associate the overproduction of NO with the cause of cancer^3^. Many synthetic, anti-inflammatory, non-steroidal drugs are used to treat inflammation in human body, but long-term use of these anti-inflammatory drugs showed severe cardiovascular and gastrointestinal problems^4^. Therefore, attention is needed for the development of new drugs as effective inhibitors of NO production in relative treatment of inflammatory diseases with fewer or no side-effects.

As science progresses the interest of researchers are shifting more towards natural products to identify new bioactive substances with better anti-inflammatory activities. The natural bioactive substances proved very helpful to replace harmful synthetic drugs^5^. In nature plants are the most interesting candidates for the presence of promising therapeutic compounds and are used to treat diseases from time immoral but most of the plants still need to be explored. Medicinal plants contain various active chemical components produced by various metabolic processes due to some external and internal factors. Each plant species has its own metabolome that manages the occurrence of chemical components. Most of these chemical components are bioactive molecules having various therapeutic potential such as antimicrobial, antitumor, anti-inflammatory and antioxidant. The ethnomedicinal uses of plants to treat pain and inflammation-related diseases are often used as a motivation for the exploration of the healing effectiveness and safety of the plant species. Many anti-inflammatory drugs such as quinine, morphine and cocaine were isolated from plants^6^.

The ability of plant extracts to modulate NO synthesis and release has been reported in previous studies on lipopolysaccharide (LPS) stimulated macrophages RAW 264.7 cells. Macrophages are one of the main components of the human immune system and play an important role by providing an abrupt defense against foreign agents prior to leukocyte migration and production of several pro-inflammatory intermediaries including free radical NO. Lipopolysaccharide is a component from the cell walls of gram-negative bacteria and is a most powerful activators of macrophages. Therefore, LPS-stimulated RAW cells are one of the potential methods to screen the anti-inflammatory drugs from plant extracts^7–9^.

For our present study, we have selected plant species *Saussurea heteromalla* (D. Don) Hand.-Mazz from Margallah hills, National Park, Pakistan. Ethno-botanically this plant is very important and used by the local community for the treatment of various ailments not only in the region of Margallah hills but throughout the Himalayas. Many researchers reported this plant for its uses for the treatment of various ailments such as the aerial parts are used as tonic for liver, kidney, and nervous system, as aphrodisiacs, for removing phlegm. The plant is also used for the reproductive disorder of women’s and the roots are used as tonic. The decoction of plants is used as anti-venom, anti-microbial, anti-inflammation, anti-arthritis, anthelmintic and antitussives^10–12^. The bioactivity of this plant is supported by the presence of many important phytochemicals such as chlorojanerin, arctigenin and arctiin, matairesinol which are well-known compounds for having anti-inflammatory and anti-tumor activities^13–15^.

Based on various ethnopharmacological uses in the present research we tried to explore this plant against anti-inflammatory activity in LPS-stimulated RAW-264.7 cell lines. An HPLC-based anti-inflammatory screening methodology was used for extracts obtained using solvents with different polarity. Then, the isolation and identification of compounds exhibiting significant activities is performed by means of UV/visible spectra and GC/MS. Purity check is assessed using melting point measurement and TLC. This study will be helpful later in the discovery of anti-inflammatory drug.

## Results

**Figure 1:**
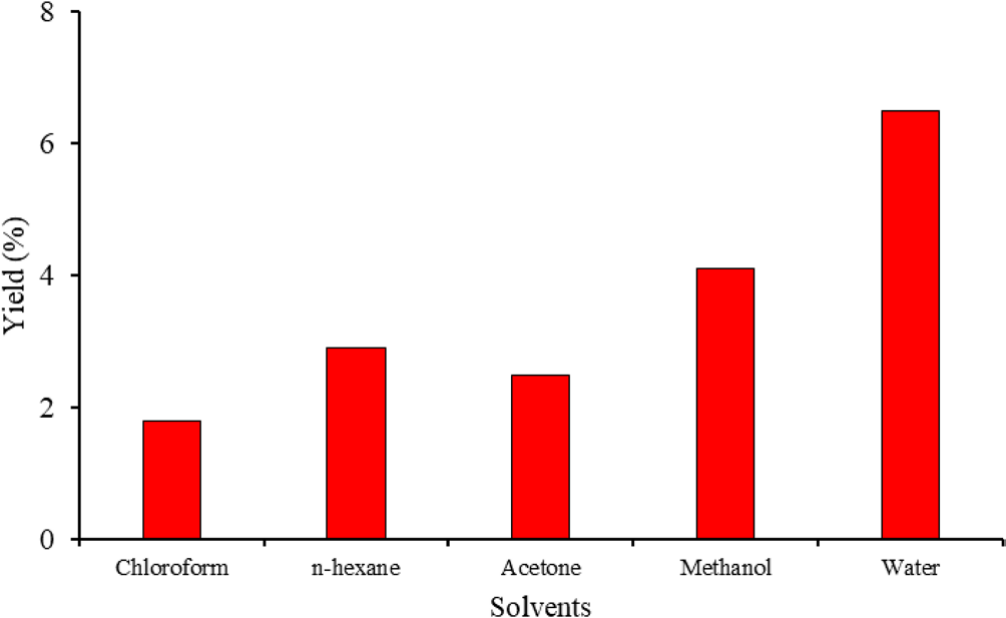
Yield percentage of *S. heteromalla* in different solvents.

*Saussurea heteromalla* underwent small-scale extraction using various solvents (*n*-hexane, chloroform, acetone, methanol, and water) to assess their efficacy in scavenging nitric oxide. The aim was to identify the most potent extract for large-scale extraction, considering both effectiveness and yield. Percentage yield was calculated using a formula, revealing that water extracts yielded the highest percentage at 6.50%, followed by ethanol extracts at 4.10%. Conversely, the chloroform extract exhibited the lowest yield, amounting to 1.80% during the small-scale extraction process (Fig. 1).

The extract from small scale extraction was then subjected for quantitative estimation of phenolic and flavonoids. Phenolic and flavonoids were estimated with the help of Linear equation of Gallic acid and Quercetin calibration curve. A considerable amount of phenolic and flavonoids were observed in all fractions. The results are expressed μg GAE/mg and μg QE/mg, data of both experiments was represented in (Table 1). In our study, phenolic and flavonoids were observed in non-polar fractions (*n*-hexane) with TPC of 123.39 ± 1.88 μg GAE/mg and TFC of (47.35 ± 2.27 μg QE/mg).

**Table 1:** Total flavonoid and phenolic content of *S. heteromalla* fractions.

SH fractions	Total phenolic (μg GAE/mg)	Total flavonoids (μg QE/mg)
*n*-Hexane	123.39 ± 1.88^a^	47.35 ± 2.27^b^
Chloroform	119.75 ± 4.03^a^	44.34 ± 4.62^b^
Acetone	98.86 ± 1.98^b^	37.56 ± 3.21^b^
Methanol	103.43 ± 1.34^b^	21.16 ± 1.76^c^
Water	102.47 ± 4.15^b^	58.07 ± 3.40^a^

The values represent the averages (± SE) of three independent replicates followed by different letters within columns are significantly different at P ≤ 0.05, according to Least Significant Difference test.

**Figure 2:**
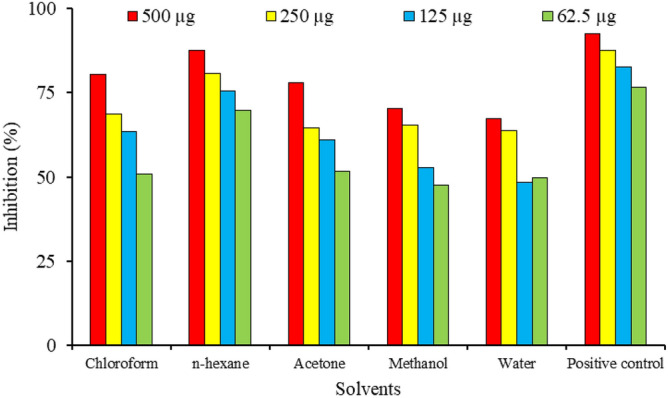
Inhibition (%) of nitric oxide by diluted fractions of plant extracts.

The high amount of Nitric oxide causes acute or chronic inflammation which some time leads to some types of cancer. So, the hyperproduction of NO should be suppressed in the body. Mouse leukemic macrophage (RAW 264.7) cell line is used to test the inhibition of nitric oxide by different plant extracts. The Assay was carried out in 96 well plate with the help of Griess reagent. During this study it was observed that our plant extracts at different concentrations (1000–62.5 μg) showed high to moderate inhibition of nitric oxide in RAW 264.7 macrophage cell line (Fig. 2). The high inhibition activity was observed in *n*-hexane fraction.

**Figure 3:**
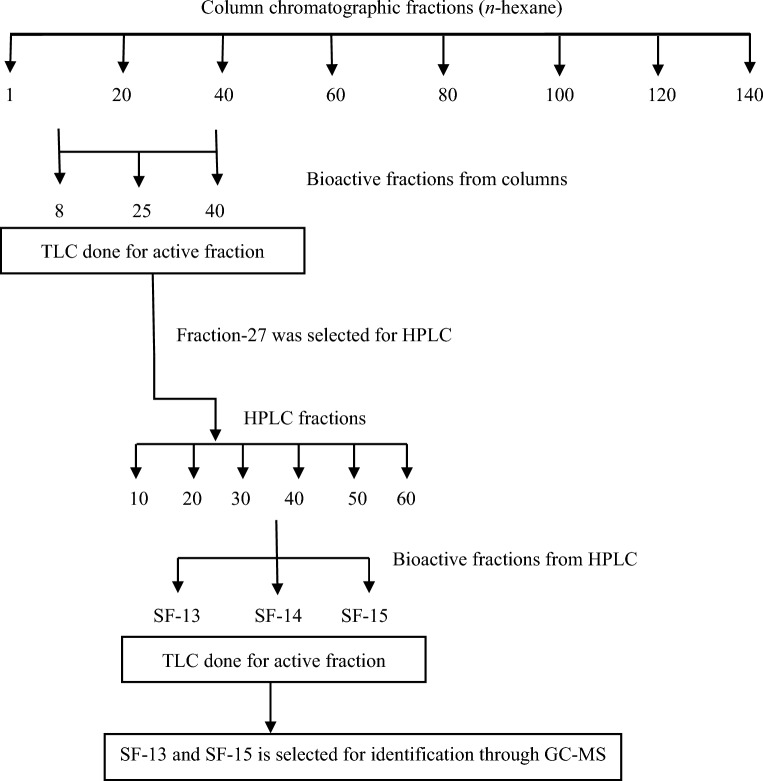
Summary of the fractionation for the compounds.

**Figure 4:**
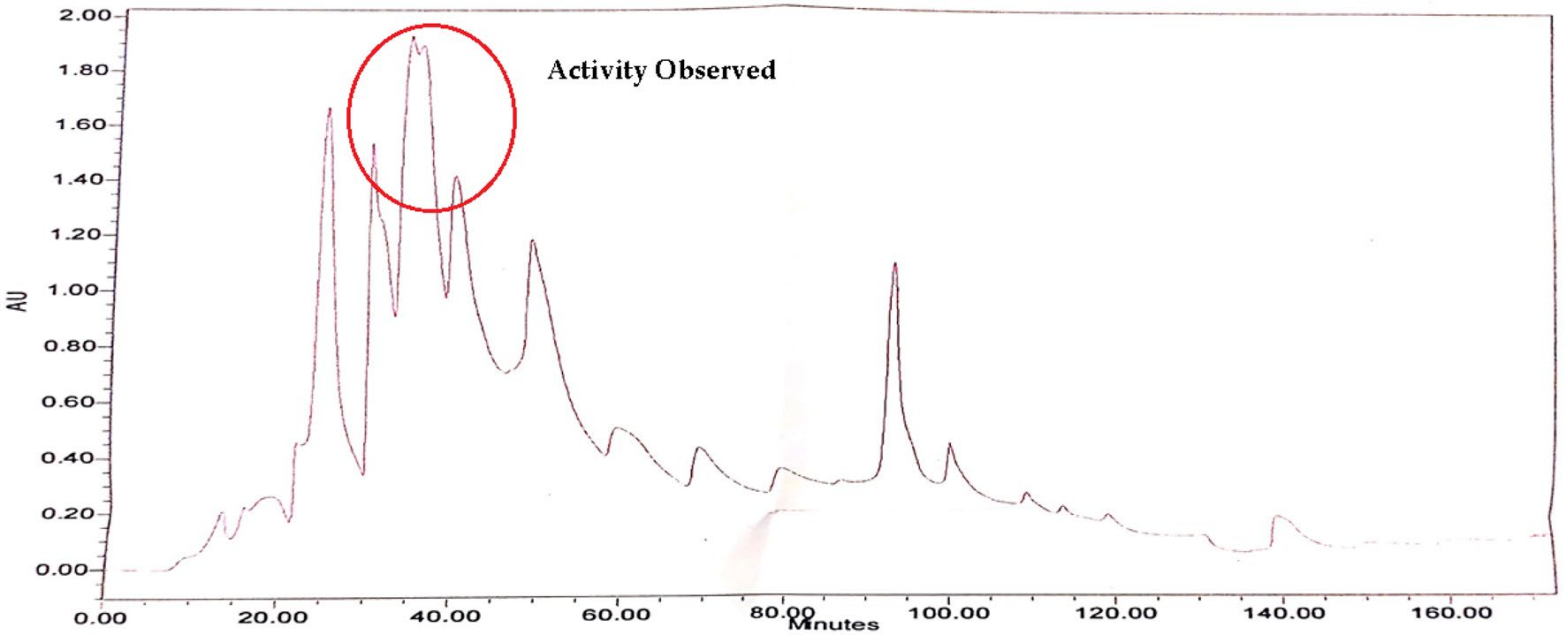
Absorption spectra from column chromatography at wavelength of 254–280 nm.

Thus, *n*-hexane extract was selected for bioassay-guided purification. Successive large-scale extraction was done with *n*-hexane solvent (Fig. 3). 8.63 g of *n*-hexane fraction was run in column chromatography for isolation, 125 different fractions were obtained (Fig. 4).

All 125 different fractions were subjected to a nitric oxide scavenging assay, in which fraction 8–32 showed activity. All fractions from column were subjected to TLC, because TLC represents first source of information for the identification and separation of compounds and is most reliable in identification of purity of the tested substance^16^. Based on TLC results fraction 27 (85% hexane and 15% dichloromethane) was found most potent for further evaluation.

**Figure 5:**
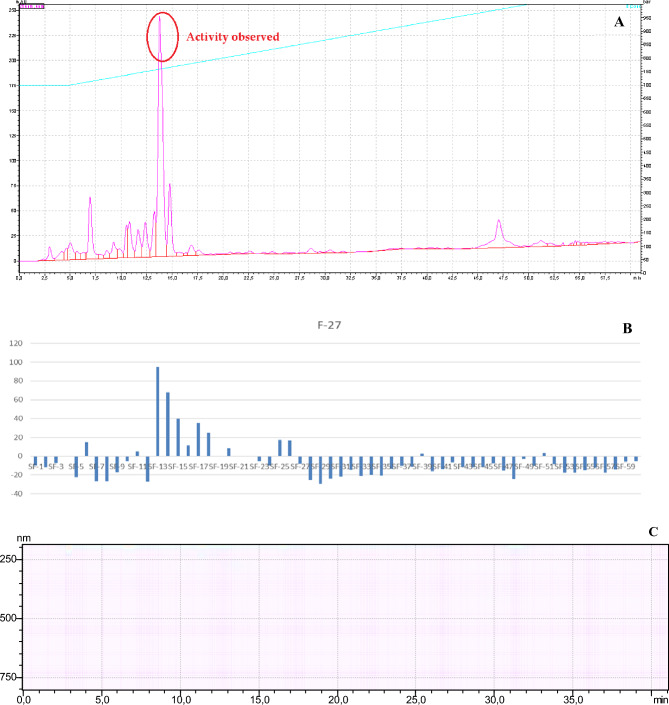
(**A**) HPLC–DAD absorption spectra of column chromatographic farction-27 and (**B**) nitric oxide scavenging ability (C) x-ray chromatogram.

Fractions F-27 were further separated with the help of HPLC–DAD. Analysis for F-27 was done with 90% acetonitrile and 10% water, 60 different sub-fractions were obtained and all these fraction were subjected to nitric oxide inhibitory activity. In F-27 sub-fractions SF-13 and SF-15 showed inhibition of NO in RAW 264.7 cell line where in all other fractions the high amount of NO is observed. In HPLC chromatogram active peaks were observed on SF-13 and SF-15 (Fig. 5) and presence of compound in x-ray chromatogram. TLC was used to test the purity of sub-fraction. On the base of TLC sub-fraction SF-13 and SF-15 were selected for further analysis and collected at the amount of 2.9 mg and 2.4 mg for GC–MS analysis.

**Figure 6:**
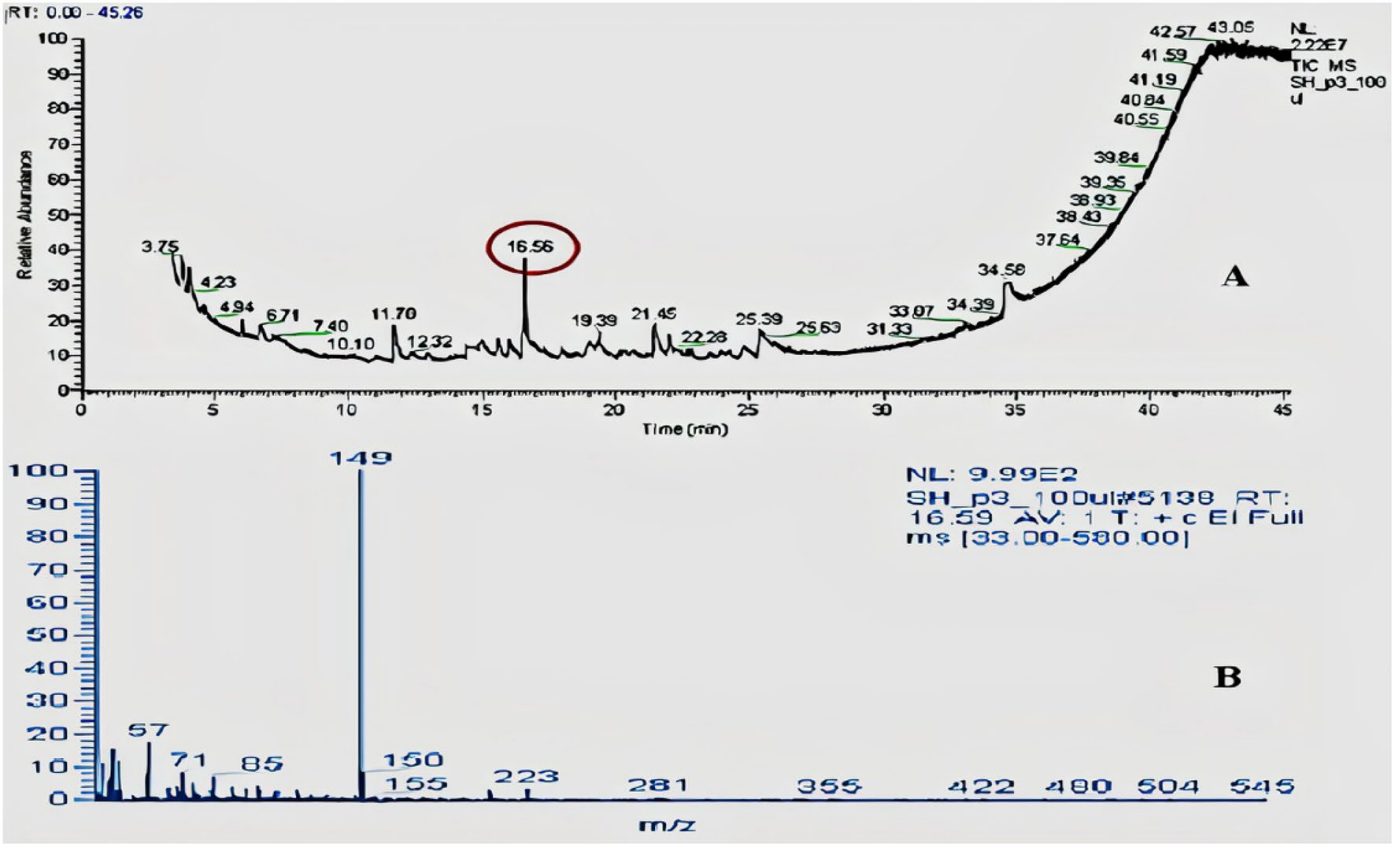
(**A**) GC–MS absorption spectra spectrum of SF-13. (**B**) MS spectrum of observed compound.

**Figure 7:**
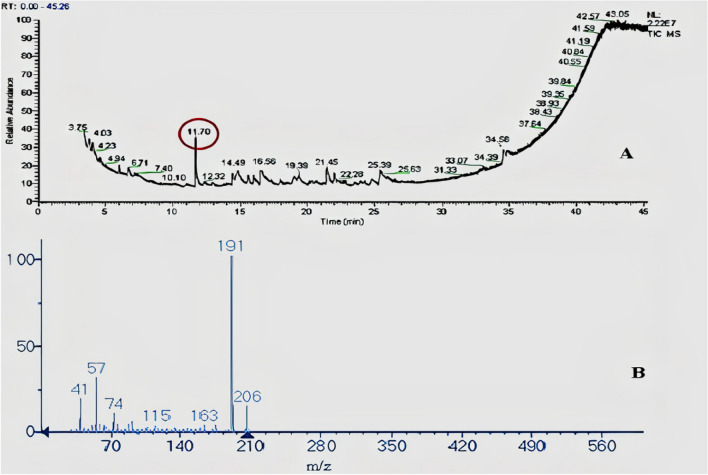
(**A**) GC–MS absorption spectra spectrum of SF-15. (**B**) MS spectrum of observed compound.

**Figure 8:**
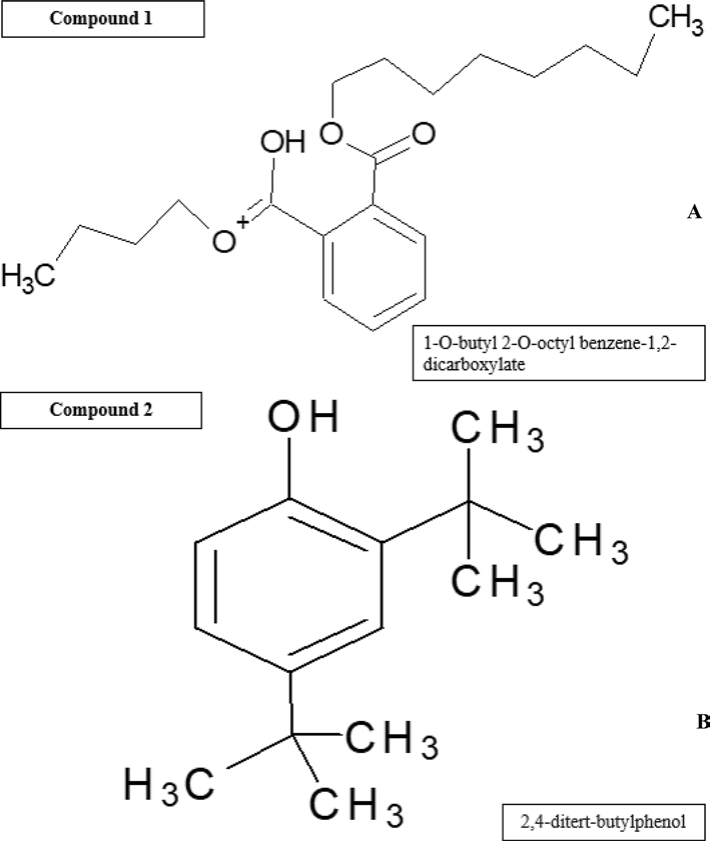
Identified compounds and their structure (**A**) 1-O-butyl 2-O-octyl benzene-1,2-dicarboxylate, (**B**) 2,4-ditert-butylphenol.

When SF-13 was subjected to GC–MS analysis, In MS spectra of GC–MS graph the retention time of compound peak was 16.56 min. while the area percentage (%) of peak was 0.62. The MS graph of this compound showed the highest peak at 149 m/z (Fig. 6). Physically this compound was an oily liquid with a boiling point of 378.1 °C while the density of the compound was 1.01 g/cm^3^. SF-15 was when subjected to GC–MS analysis the retention time of the compound was 11.70 min. The MS graph of this compound showed the highest peak at 191 m/z (Fig. 7). Physically this compound was solid with a boiling point of 263.7 °C. Both compounds were analyzed with NIST data base, NIST identified compound one as 1-O-butyl 2-O-octyl benzene-1,2-dicarboxylate while compound 2 was identified as 2,4-ditert-butylphenol (Fig. 8).

## Discussion

Plants always played an important role in the discovery of new valuable healing agents and received significant focus because of the presence of important secondary metabolites. These secondary metabolites played important roles as antioxidants, anti-microbial, anti-inflammatory, and anti-tumor. WHO (world health organization) reported more than 20,000 plant species, used for the treatment of different ailments worldwide. These medicinal plants are rich in terms of secondary metabolites which play an important role as therapeutic agents and display properties of anti-tumor, antioxidant, anti-microbial and anti- inflammatory. Phenolic compounds are one of the widespread secondary metabolites in plants, they serve an important role in flower pigmentation and act as protecting agents against attacking organisms. For the bioactivities they are reported by many researchers for their vast therapeutic potential in human body such as, anti-inflammatory, antioxidant, anti-microbial and anti-tumor activities.

In our present study different solvents (methanol, water, chloroform, acetone, and *n*-hexane) were used for the extraction of *S. heteromalla*. Different solvents showed different percentage yield high yield was observed in polar solvents. The variation in yield percentage was due to the reason that some phytochemicals have different solubility in polar and non-polar solvents. Mostly the *n*-hexane shows lowest yield due to lower polarity while the ethanol and water extract shows high yield due to high polarity. It has been reported by researchers that water and ethanolic extracts of plants have high yield as compared to other solvents, due to their high polarity and high absorption for polar substances^17–19^.

However, the quantitative estimation of the phenolic and flavonoids in our case were found most abundantly in non-polar, *n*-hexane fraction and this fraction is also observed to exhibit high nitric oxide scavenging ability. The quantitative presence of secondary metabolites is different in different plant species. The researchers described the presence of these secondary metabolites are due to the response of plants to their environmental condition. So, for each plant the quantity of these secondary metabolites varies. Phenolic and flavonoids are the most important and biologically active ones. The biological activity of a plant depends on the quantity of these phytochemicals^20,21^.

Most of the research prove the strong relationship of these secondary metabolites especially polyphenols with the free radical scavenging and anti-inflammatory activities, some of polyphenols like, glutathione, α-tocopherol, quercetin, hydroquinone, curcumin, resveratrol, capsaicin and epigallocatechin-3-gallate are utilizing at commercial level as free radical scavenger and anti-inflammatory compounds to protect the body^22^. During in vitro evaluation of *S. heteromalla* fraction the *n*-hexane fraction showed the high scavenging ability of nitric oxide as compared to other fractions. The high scavenging of *n*-hexane will be associated with the high level of phenols and flavonoids^23,24^. Another plant from the same genus *S. lappa* showed to exhibit nitric oxide inhibition and excessive pro-inflammatory cytokine. Lee and Kang associated this activity with high levels of phenols^25^.

Bioassay guided fractionation of present study led to the isolation of two free radical scavenging compounds 2, 4-ditert-butylphenol and 1-O-butyl 2-O-octyl benzene-1, 2-dicarboxylate. The most active was 2, 4-Di-tert-butylphenol. The GC–MS spectrum of this compound showed the highest ion peak at 191 m/z and second highest at 57 m/z with the molecular formula C_14_H_22_O and molecular weight of 206.32. It is a colorless solid, with a melting point 36 °C, soluble in many organic solvents including water. 2, 4-Di-tert-butylphenol is identified as a strong antioxidant by many researchers to inhibit free radical scavenging activity^26–29^.

2,4-Ditert-butylphenol is identified in many plant species, bacteria, fungi, and some species of animals. In family Asteraceae this compound has been reported from plant species *Ageratina adenophora* and *Cyanthillium Cinereum*^30–32^. As our study report 2, 4-ditert-butylphenol as high inhibitor of free radical nitric oxide. Many researchers report this compound as a good antioxidant but apart from antioxidant activity this compound possesses antibacterial, anti-inflammatory, anti-tumor and possess larvicidal activity^33,34^.

The second most active compound of our study was 1-O-butyl 2-O-octyl benzene-1,2-dicarboxylate ester also known as phthalic acid butyl octyl ester, it is a colorless liquid with a low volatility, with the boiling point of 370 °C, soluble in organic solvents and oils but have poor solubility in water^35^. The GC–MS spectrum of this compound showed the highest ion peak at 149 m/z with the molecular formula of C_20_H_30_O_4_ and molecular weight of 334.45. This compound is well known for its microbial and insecticidal activity. In industries it is commonly known for its plasticizers activities^36,37^.

Literature surveys revealed that naturally, this compound occurs in many plant families but most commonly in Lamiaceae, Rosaceae, Solanaceae, Liliaceae, and Asteraceae. From Asteraceae family three plant species *Ageratina adenophora, Cirsium japonicum* and *Chrysanthemum indicum* have been reported by different research for having phthalic acid butyl octyl ester in different parts^38,39^. Different researchers reported this compound for its antimicrobial effect against bacteria and fungi and in some cases, it reduces the damage caused by these pathogens^40,41^. In our study the phthalic acid butyl octyl ester showed moderate inhibition of nitric oxide scavenging. Previously it has been reported from *Aloe vera* extracts and possesses high antioxidant activity^42^.

## Materials and methods

### Collection and identification of plant material

**Figure 9:**
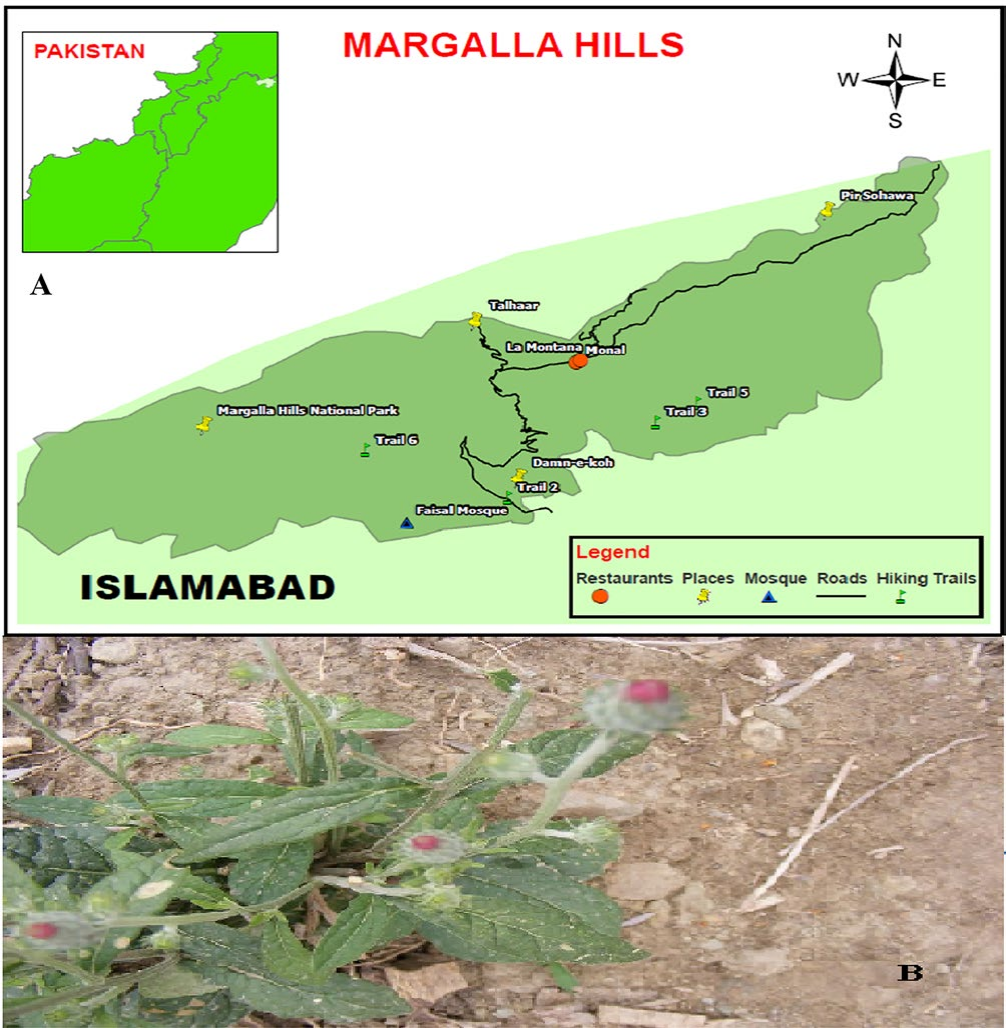
(**A**) Map of the study area, (**B**) Selected plant species.

The whole Plant, *S. heteromalla,* was collected from the Margallah Hills National Park, Pakistan (Fig. 9) and authenticated using reference materials from the herbarium of Quaid-i-Azam University, Islamabad, Dr. Muhammad Zafar identified the specimen. For the record the specimen with voucher number ISL-798 was deposited. After that with the permission from the administration of Quaid-i-Azam University, Islamabad, we started the collection of the plant samples. To preserve its color and volatile compounds, the plant was dried naturally at room temperature and stored in the dark. The dried material was then pulverized to fine powder before extraction and stored at 4 °C until extraction. Our study complies with relevant institutional, national, and international guidelines and legislation.

### Small-scale extraction

Small-scale extractions were performed by using the method of^43^. 1 g of powder plant material was taken separately into four 15 mL sterile Falcon tubes and extracted with the solvents of different polarity: hexane, chloroform, acetone, methanol, and water. The tubes were left standing for 24 h, during this time the tubes were sonicated 4 times into bath sonicator (Branson B5510) for 60 min. Each sample was centrifuged at 2000 rpm for 20 min. The supernatant was transferred to smaller tubes. 1 mL from each extract was transferred into a 2.5 mL polyvinyl chloride Eppendorf tube and evaporated in a Savant Speedvac Concentrator. The dried organic solvent residue was re-dissolved in DMSO, and aqueous extract was re-dissolved in water to prepare the stock of 20 mg/mL. The samples were then stored at 4 °C till bioactivity testing.

### Total phenolic content

The total phenolic contents were measured by Folin–Ciocalteu reagent calorimetric method^44^ with some modifications. 1 mg plant extract was dissolved in 1 mL methanol (Sigma-Aldrich, USA). Gallic acid (Sigma-Aldrich, USA) was used as a standard with the range of dilutions (15.62–500 µg). From each extract and control 300 μL was mixed with 2.5 mL Folin-Ciocalteu phenol reagent (Merck, New jersey, USA), after 5 min. 2.5 mL, 6% Sodium Carbonate (Sigma-Aldrich, USA) was added into it. The mixture was then incubated for 90 min. at room temperature. After which the absorbance was measured at 725 nm. Standard calibration curve was prepared for gallic acid and the total phenolic of plant extracts was calculated form that calibration curve as mg Gallic acid equivalent per gram (mg GAE/g).

### Total flavonoids content

Total flavonoid contents were measured with the help of aluminum chloride colorimetric method^45^ with some modifications. 1mg plant extract was dissolved in 1 mL methanol. Quercetin (Sigma-Aldrich, USA) was used as a Standard, standard calibration curve was developed from different dilution (15.62–500 µg) of quercetin. 500 μL of each plant extract and control was taken with 1.5 mL of methanol, 100 μL of 10% aluminum chloride solution (Merck, New jersey, USA), 100 μL of 1 M Potassium acetate solution (Merck, New jersey, USA) and 2.8 mL of distilled water to make the total volume up to 5 mL. The mixture was then incubated at room temperature for 30 min. Absorbance was taken at 415 nm. From the Standard calibration curve of Quercetin results of all plant extracts were measured as mg Quercetin equivalent per gram (mgQE/g).

### Large-scale extraction (successive)

200 g of the dried plant powder was transferred to a 2.5 L glass bottle; 1 L hexane was added and thoroughly mixed with the plant powder. The bottle was placed in a sonication water bath four times, 60 min. each after every 6 h to improve extraction. Then, the extract was filtered by 185 mm filter paper and evaporated on a rotary evaporator (BUCHI). The process was repeated until the extract yield became insignificant. The fraction was concentrated under reduced pressure using a rotary evaporator^43^.

### Nitric oxide scavenging assay

The nitric oxide scavenging assay utilized RAW 264.7 cell lines, known as mouse leukemic macrophages. These cells were stimulated using Lipopolysaccharides (LPS) derived from *E. coli* to induce the synthesis of nitric oxide.

#### Cells culture

Cell line was obtained from the animal physiology and neurobiology lab, zoological department, KU Leuven. Thawing was done from liquid nitrogen stock. After thawing the cells were transferred to sterile falcon tube and 5 mL of cell culture media was added to it. After observing the density of cell with the help of microscope the 2 mL of cell culture was transferred to the cell culture flask and 15 mL Dulbecco’s Modified Eagle’s Medium (DMEM) (supplemented with 10% fetal calf serum (FCS) and 5 μg/mL of Penicillin/Streptomycin/Fungizone (PSF) was added to the flask. After that the flask was kept at 37 °C for 24 h in incubator at 5% CO_2_ to let the cell adhere to the surface of the flask and grow^2^.

#### Experiment

The Griess colorimetric reaction method was utilized for the assay ^6^. After 48 h, cell density was assessed. Upon observation, the cultured cells were rinsed, detached, and then placed in 96-well microtiter plates (2 × 10^5^ cells per well), counted using a cell counter. Subsequently, 100 μL of media was added and the plates were incubated at 37 °C overnight to allow the cells to adhere. Once the cells were firmly attached after 24 h, they were exposed to 10 μL of plant extracts, along with positive and negative solvent controls. Following a 2 h incubation at 37 °C with the test extracts, 50 μL of LPS (5 µg/mL) in DMEM was introduced into all the wells and further incubated for 24 h. Post-incubation, supernatants from the cells were collected for nitric oxide measurement. In a 96-well plate, 100 μL of cell supernatant was mixed with an equal volume of Griess reagent (Sigma-Aldrich) and kept at room temperature for 10 min. Subsequently, absorbance was measured at 550 nm using a plate reader (FlexStation® 3 Multi-Mode Microplate Reader, SoftMax® Pro Software, version 4.3.1) to determine the percentage inhibition of nitric oxide.

### Bioassay-guided purification

8.63 g of dried active hexane extract were adsorbed onto 30 g of silica from Sigma-Aldrich. A sufficient amount of silica gel was mixed with petroleum ether to form a slurry, which was then loaded onto a column measuring 35 × 500 mm with a pressure of 40 bars, ensuring the exclusion of any air bubbles. A dried sample absorbed on silica was positioned at the column's top. Subsequently, the column underwent elution using a step gradient that increased in polarity: hexane:dichloromethane ratios of 9.5:0.5, 9:1, 8.5:0.5, 8:2, 7:3, 6:4, 5:5, 4:6, 3:7, 2:8, 1:9, and 0:10. This was followed by elution with 100% dichloromethane, then 100% ethyl acetate, and subsequently, a series of ethyl acetate and methanol mixtures in ratios of 9.5:0.5, 9:1, 8:2, 7:3, 6:4, 5:5, 4:6, 3:7, 2:8, and 1:9. Finally, the column was flushed with 100% methanol. At each stage, ten tubes of 40 mL fractions were collected, and the solvent was evaporated. The entire separation process was monitored using a Dual λ model 2487 absorbance detector at wavelengths of 280 nm and 254 nm^5^.

#### HPLC–DAD analysis

HPLC analyses were conducted using a Shimadzu LC-20AT system quaternary pumps, paired with SPD-M20A photodiode array detector, CBM-20A/20A interface, Japan. Software: LC Solution. Lab Solution software version 10.0.1was employed for data acquisition and processing. The active fraction 11 underwent analysis utilizing a reverse-phase HPLC column: SunfireTM prep C18 (10 mm × 250 mm, 5 μm). The mobile phase consisted of 10% H2O and 90% acetonitrile (LC–MS CHROMASOLV®, Fluka), supplemented with 0.1% TFA (ACROS ORGANICS). Before HPLC injection, samples were filtered through a CHROMAFIL® Xtra H-PTFI filter. A two-milliliter sample was injected and run for 60 min. at 20 °C with a flow rate of 4 mL/min^5^.

#### GC–MS analysis

The GC–MS analysis of various extracts was conducted using a Shimadzu QP2010 system, which includes an auto-sampler high-performance gas chromatograph coupled to a Mass Spectrometer. This analysis utilized a capillary column (Rtx®-5Ms) with dimensions of length 30 mm, inner diameter 0.25 mm, and a thickness of 0.25 µm. Chromatographic conditions involved an initial temperature of 80 °C, a final temperature of 280 °C, and an injection temperature of 250 °C. The column was initially set at 80 °C for 2 min, followed by a gradual increase to 280 °C at a heating rate of 5 °C per min, maintaining this temperature for 12 min. Helium gas (99.99%) served as the carrier gas, flowing at a constant rate of 1 mL/min. GC–MS data was analyzed using NIST database software to cross-reference and correlate the detected compounds with available data in the database.

### Data analysis

Data obtained was analyzed for analysis of variance (ANOVA) by two-factor factorial in Completely Randomized Design (CRD) and mean comparisons (p ≤ 0.05) were carried out by Least Significant Difference (LSD) test using M-Stat-C Statistical software^46^.

## Conclusions

In conclusion, the *n*-hexane extracts of *S. heteromalla* leaves showed free radical scavenging activity due to the presence of 1-O-butyl 2-O-octyl benzene-1,2-dicarboxylate and 2,4-ditert-butylphenol. This supports the traditional medicinal use of this plant for the treatment of different aliments including anti-inflammation and antioxidants. Due to presence of antioxidant compounds the plant extracts can be used as a natural source of antioxidant as they showed the promising scavenging of free radicals NO. The literature study of these compounds also confirmed their importance as antioxidants. Therefore, it is advised to use the plant extract as a natural source of antioxidants at industrial level after checking their cytotoxicity. So, it is suggested that isolated compounds should be studied against different biological activities and cell lines to identify their other potential activities and toxicity. Detailed study is suggested to understand its mode of action and possible synergistic effects with other compounds.

## Data Availability

The datasets used and/or analyzed during the current study are available from the corresponding author on reasonable request.
